# Population pharmacokinetic modelling of once-daily ritonavir-boosted darunavir in HIV-infected patients

**DOI:** 10.1186/1758-2652-13-S4-P184

**Published:** 2010-11-08

**Authors:** L Dickinson, AGA Jackson, LJ Garvey, V Watson, SH Khoo, A Winston, M Boffito, GR Davies, DJ Back

**Affiliations:** 1 NIHR Biomedical Research Centre, Royal Liverpool & Broadgreen University Hospital, Liverpool, UK; 2 Chelsea & Westminster Hospital, SSAT, London, UK; 3 Imperial College, London, UK; 4 University of Liverpool, Department of Pharmacology, Liverpool, UK; 5 St Stephen's Centre, Chelsea & Westminster Foundation Trust, London, UK

## Purpose of the study

Once daily ritonavir-boosted darunavir (DRV/RTV) is a preferred antiretroviral regimen for treatment-naïve patients. The pharmacokinetics (PK) of DRV/RTV may be influenced by patient demographics and co-medications. Also with an increased aging HIV population, investigations into the impact of older age on PK are important.

## Methods

Data were pooled from 3 DRV/RTV PK studies. In total 51 HIV-infected patients (7 female) stable on DRV/RTV (800/100mg or 900/100mg once daily; n=32 and 19, respectively) were included. Median age, weight, BMI, RTV area under the curve over 24h (AUC_0-24_) and baseline CD4 cell count were 39yr (21-63), 74kg (57-105), 24kg/m^2^ (18-31), 4.35mg.h/L (2.27-10.99) and 500cells/mm^3^ (227-1129), respectively; 49 patients had undetectable viral load at time of study. PK sampling was performed at steady-state and between 1-3 PK curves were available per patient. Nonlinear mixed effects modelling (NONMEM v. VI 2.0) was applied to determine DRV PK parameters, interindividual and interoccasion variability and residual error. The following covariates were evaluated: age, weight, BMI, sex, ethnicity, RTV AUC_0-24_ and raltegravir co-medication (400mg twice daily). The model was validated by means of simulation and visual predictive check.

## Summary of results

A 2-compartment model with first-order absorption (k_a_ 0.914h^-1^) and lag-time (0.358h) best described the data. Inclusion of a different apparent oral clearance (CL/F) and volume of distribution (V2/F) for one of the studies improved the fit (Study 1,2 *vs.* Study 3 CL/F: 12.5 *vs.* 15.6L/h; V2/F 125 *vs.* 192L). RTV AUC_0-24_ and age were significantly associated with DRV CL/F. An increase in age of 1yr produced a fractional decrease in DRV CL/F of 0.014, equivalent to a 14% reduction in CL/F with every 10yr increase in age. Based on the visual predictive check 94% of observed DRV concentrations were within the 95% prediction interval, indicative of an adequate model.

## Conclusions

A population model describing the PK of once daily RTV-boosted DRV has been developed and validated. RTV AUC_0-24_ and age were significantly related to DRV CL/F. The impact of age requires further investigation and clarification over a wide age range, particularly in an elderly population.

